# The Effect of Artificial Intelligence on Patient-Physician Trust: Cross-Sectional Vignette Study

**DOI:** 10.2196/50853

**Published:** 2024-05-28

**Authors:** Anna G M Zondag, Raoul Rozestraten, Stephan G Grimmelikhuijsen, Karin R Jongsma, Wouter W van Solinge, Michiel L Bots, Robin W M Vernooij, Saskia Haitjema

**Affiliations:** 1 Central Diagnostic Laboratory University Medical Center Utrecht Utrecht University Utrecht Netherlands; 2 Utrecht University School of Governance Utrecht University Utrecht Netherlands; 3 Julius Center for Health Sciences and Primary Care University Medical Center Utrecht Utrecht University Utrecht Netherlands; 4 Department of Nephrology and Hypertension University Medical Center Utrecht Utrecht Netherlands

**Keywords:** patient-physician relationship, trust, clinical decision support, artificial intelligence, digital health, decision support system

## Abstract

**Background:**

Clinical decision support systems (CDSSs) based on routine care data, using artificial intelligence (AI), are increasingly being developed. Previous studies focused largely on the technical aspects of using AI, but the acceptability of these technologies by patients remains unclear.

**Objective:**

We aimed to investigate whether patient-physician trust is affected when medical decision-making is supported by a CDSS.

**Methods:**

We conducted a vignette study among the patient panel (N=860) of the University Medical Center Utrecht, the Netherlands. Patients were randomly assigned into 4 groups—either the intervention or control groups of the high-risk or low-risk cases. In both the high-risk and low-risk case groups, a physician made a treatment decision with (intervention groups) or without (control groups) the support of a CDSS. Using a questionnaire with a 7-point Likert scale, with 1 indicating “strongly disagree” and 7 indicating “strongly agree,” we collected data on patient-physician trust in 3 dimensions: competence, integrity, and benevolence. We assessed differences in patient-physician trust between the control and intervention groups per case using Mann-Whitney *U* tests and potential effect modification by the participant’s sex, age, education level, general trust in health care, and general trust in technology using multivariate analyses of (co)variance.

**Results:**

In total, 398 patients participated. In the high-risk case, median perceived competence and integrity were lower in the intervention group compared to the control group but not statistically significant (5.8 vs 5.6; *P*=.16 and 6.3 vs 6.0; *P*=.06, respectively). However, the effect of a CDSS application on the perceived competence of the physician depended on the participant’s sex (*P*=.03). Although no between-group differences were found in men, in women, the perception of the physician’s competence and integrity was significantly lower in the intervention compared to the control group (*P*=.009 and *P*=.01, respectively). In the low-risk case, no differences in trust between the groups were found. However, increased trust in technology positively influenced the perceived benevolence and integrity in the low-risk case (*P*=.009 and *P*=.04, respectively).

**Conclusions:**

We found that, in general, patient-physician trust was high. However, our findings indicate a potentially negative effect of AI applications on the patient-physician relationship, especially among women and in high-risk situations. Trust in technology, in general, might increase the likelihood of embracing the use of CDSSs by treating professionals.

## Introduction

### Background

It was John McCarthy who coined the term “artificial intelligence” (AI) at the Dartmouth conference in 1956 and defined it as “the science and engineering of making intelligent machines, especially intelligent computer programs” [1]. However, it was only in the 1990s, after the first so-called “AI winter,” that interest in AI began to increase again [2]. Since then, AI applications have been on the rise, ranging from self-driving cars to AI-powered web search [3]. Development and implementation of AI in health care is similarly increasing. It is believed that AI has the potential to improve every facet of health care—screening, diagnosis, prognosis, and treatment [4]. Although the use of AI in routine clinical care is still in the early stages, it has already shown promise in specific medical fields, such as radiology, for the recognition of complex patterns in imaging data [3,5]. Hospital-wide strategic programs have been initiated to develop predictive AI algorithms based on routine clinical care data in various hospitals [6]. The goal of these projects is, among others, to integrate AI algorithms in clinical decision support systems (CDSSs). CDSSs are often classified as either knowledge-based or non–knowledge-based systems. Knowledge-based CDSSs provide an output by evaluating a certain rule, which is programmed based on evidence or practice. Non–knowledge-based CDSSs use AI techniques, such as machine learning, for decision support and prediction [7]. AI-based CDSSs are often developed when dealing with complex, high-dimensional, and large amounts of data (ie, big data), such as routine care data. By linking patient information to evidence-based knowledge, a CDSS can provide case-specific information, which may support physicians in developing more personalized judgments and recommendations [8]. Despite these efforts, however, to date, only a fraction of all developed AI-based algorithms have been implemented in clinical care [9].

**Figure 1:**
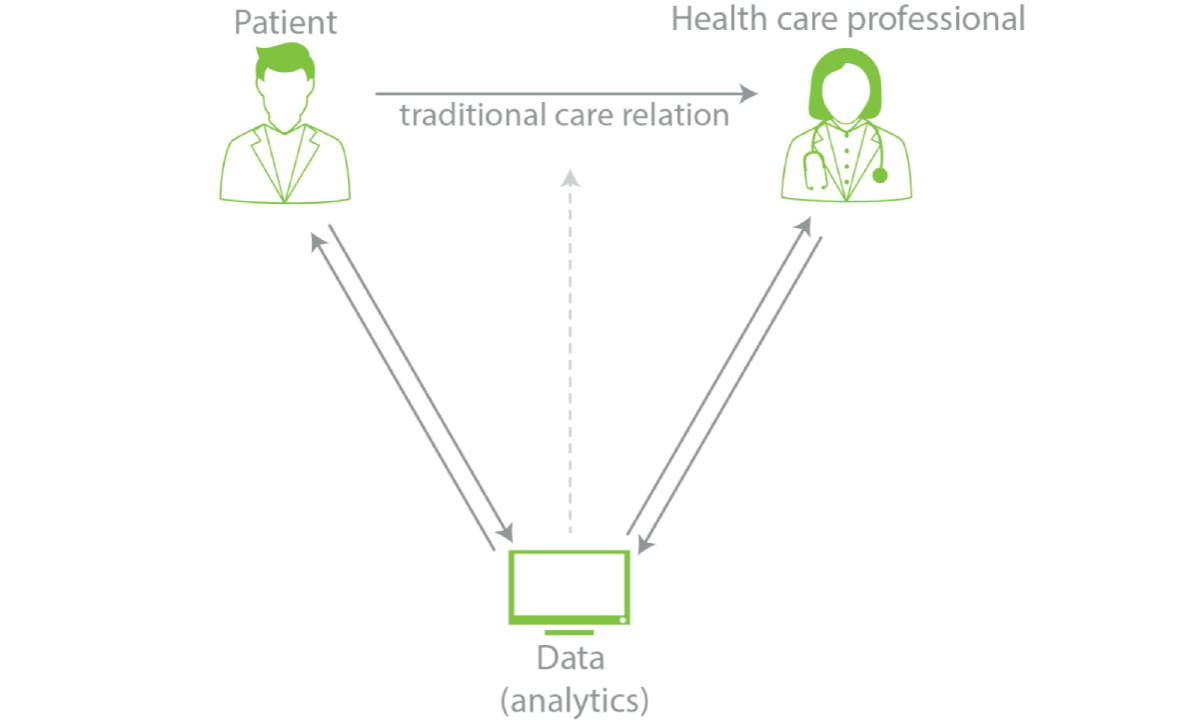
Relationships between the patient, health care, and data (adapted from Groenhof [17], which is published under Creative Commons Attribution 4.0 International License [18]).

The debate on AI-based CDSSs in health care has mainly focused on the technical aspects of the technology [10,11], including questions like “How well does the predictive algorithm perform in terms of, for example, recall and precision?” or “What is the importance of its features?” To date, less attention has been given to the acceptability of using AI-based CDSSs in terms of patients’ or physicians’ trust in CDSSs even though these are crucial for the implementation and acceptance of these systems [12,13]. Some concerns revolve around how the patient-physician relationship is directly affected by the integration of AI applications into clinical practice. This concern arises due to all the new possibilities that AI offers, such as decision support, patient dashboards, and eHealth [14-16]. Figure 1 illustrates how the relationship between a patient and physician could be influenced by AI [17,18]. Studies have shown that algorithms developed to diagnose or predict a disease often perform as well as, and sometimes even better than, a physician [19]. As a result, physicians may be able to make more informed decisions and subsequently improve patient care, which was the incentive to start the Applied Data Analytics in Medicine (ADAM) project at the University Medical Center (UMC) Utrecht [6]. On the other hand, to build and maintain interpersonal trust, patient involvement in the decision-making process is important [20-23]. However, these algorithms, especially those that include AI, are sometimes perceived as “black boxes,” which could potentially lead to a reduction in trust in the physician, even though these algorithms can be beneficial to the patient’s care process. In medicine, trust is considered a central aspect of the patient-physician relationship. Without trust, treatments have proven to be less effective, and patients are more prone to ask for a second opinion [24].

### Trust

To the best of our knowledge, we are among the first to study the impact of AI-based CDSSs on patient-physician trust in both a clinical high-risk and low-risk setting. Trust is a complex and multidimensional concept [25]. Over the past decades, a lot of research has been conducted on this notion and various dimensions of trust have been described in the academic literature [26]. The most commonly named dimensions of trust in a physician are privacy and confidentiality [27,28], compassion [27,29], reliability and dependence [28], competence [27-31], communication [32], and honesty [33]. However, these dimensions of trust are often studied separately. With so many described trust dimensions, it is difficult to develop a framework specifically for patient-physician trust. Therefore, a framework that integrates these components of trust in a physician has not been established yet. In social sciences, trust is commonly studied in 3 dimensions: benevolence, competence, and integrity [34-37]. In a clinical context, benevolence is the extent to which a patient perceives a physician as caring about the patient’s personal and health interests. Competence is the extent to which a patient perceives a physician as competent, capable, effective, and professional. Integrity concerns the extent to which a patient sees a physician as honest and truthful, handling the patient’s sensitive information with care and confidentially.

We aim to study the extent to which these aspects of patient-physician trust are affected by AI-based CDSSs.

## Methods

We followed the Strengthening the Reporting of Observational Studies in Epidemiology (STROBE) guidelines for observational (cross-sectional) studies.

### Study Setting

We conducted a cross-sectional vignette study at the UMC Utrecht, the Netherlands. We reached out to all members of the UMC Utrecht patient panel (N=860) as part of the ADAM program (2017-2020; currently known as the Digital Health Department within the UMC Utrecht) [6]. In brief, the ADAM program consisted of 11 projects all aiming to answer clinical questions to personalize health care using routine care data and innovative methodology, such as AI. The overall goal of the ADAM program was to investigate the need to apply this health care innovation on a large scale at the UMC Utrecht to establish a learning health care system [6].

For our study, we simulated possible future clinical decision support applications of 2 of the ADAM projects. The Neonatal Intensive Care Unit (NICU) application concerned data of babies between 24 and 32 weeks old admitted in the NICU using an algorithm that was trained on a data set from the Wilhelmina Children’s Hospital of the UMC Utrecht. This CDSS tool aimed to predict late-onset sepsis using the algorithm to prevent unnecessary antibiotic use and enable timely treatment [6]. The rheumatoid arthritis (RA) CDSS worked on the premise that disease flares can be predicted based on data about the course of the disease, patient characteristics, and information about current treatment. The aim of the RA application was to predict flares to support data-driven reduction of high preventative doses of RA medication [6,38]. This algorithm was trained on data of patients with RA of the UMC Utrecht [38].

### Study Population

Members of the patient panel (N=860) were all current and former patients of the UMC Utrecht willing to participate in research from the UMC Utrecht. The majority (693/860, 80.6%) of the panel was between 45 and 80 years of age, 57.3% (493/860) had higher education levels, and 52.8% (454/860) of the patient panel were women.

### Questionnaire

In our study, we focused on interpersonal trust. We used the definition from Mayer et al [34] to define interpersonal trust as “the willingness of a party to be vulnerable to the actions of another party based on the expectation that the other will perform a particular action important to the trustor, irrespective of the ability to monitor or control that other party.”

We studied 3 dimensions of trust: competence, integrity, and benevolence. We used an adapted version of the “Trust in Physician Scale” to assess patient-physician trust. The “Trust in Physician Scale” is a questionnaire consisting of 11 items and is commonly used to measure patient-physician trust [28,39]. Table 1 illustrates how the 11 questions of the Trust in Physician Scale were adapted and divided into the 3 trust dimensions.

In addition to the questions of the Trust in Physician Scale, we added several self-constructed questions to study these aspects of trust more comprehensively. We added the following 3 statements in the integrity dimension:

- I have the feeling that this physician is not holding anything back from me (scale 1-7).
- I have the feeling that this physician is being honest with me (scale 1-7).
- I trust this physician is handling my medical data with care (scale 1-7).

One additional statement was added in the benevolence dimension, as follows: “This physician’s recommendation is in my personal best interest (scale 1-7).”

Besides trust in the physician, we added 2 additional questions about trust in health care and technology in general on a 7-point Likert scale (1: “no trust at all”; 7: “fully trust”), as follows:

- Please indicate your level of trust in health care in general (scale 1-7).
- Please indicate your level of trust in technology in general (scale 1-7).

Additionally, we asked the panel members their age, sex, and education level.

Subsequently, we performed a factor analysis on the questionnaire, including the Trust in Physician Scale and the self-constructed questions, to check whether the predefined dimensions were present in the questionnaire. The factor analysis revealed the correlations between the items of the questionnaire and subdivided them into factors. Thereafter, we calculated Cronbach α to measure the degree of coherence between the 3 trust dimensions [40]. A Cronbach α of more than 0.75 was considered high, and thus, acceptable, meaning that all items of the dimension measured the same concept [41].

**Table 1:** Trust in Physician Scale questions divided by dimensions of trust, rated on a 7-point Likert scale, with 1 indicating “strongly disagree” and 7 indicating “strongly agree,” as answer possibilities.

Dimension	Statement
Benevolence	This physician really cares about me as a person. This physician’s recommendation takes into account my wishes and puts them first. I trust this physician’s recommendation will put my medical needs above other interests. I feel that with this recommendation the physician does everything to help me.
Competence	This physician is a real expert in taking care of medical problems like mine. I trust this physician’s judgment about this medical problem. I trust this physician’s recommendation and therefore have no need for a second opinion. I trust my physician’s recommendation so much that I will follow it.
Integrity	I trust this physician will tell me if he/she makes a mistake in this recommendation. If this physician tells me this recommendation, then I also believe that this recommendation is correct. I feel that this physician keeps the medical information used for this recommendation private.

### Design of Data Collection

Data were collected in April 2019. The questionnaire included 1 of the 2 hypothetical cases in the form of a so-called “vignette.” These vignettes were developed together with physicians from the neonatology and rheumatology department as well as the ADAM program staff to make them as realistic as possible (eg, to assess whether the physician’s communication style in the vignette reflected clinical practice) and to ensure that the AI-based CDSS applications of both projects were accurately described. The vignettes were tested on master’s degree students of Public Management from Utrecht University and employees of the ADAM program (N=36) first to study whether they could empathize well with the situation presented to them. This test resulted in a mean score of 4.6 for the NICU and 5.7 for the RA vignette on a 7-point Likert scale (the higher the score, the better they could empathize with the situation presented). We processed the feedback we received from the test participants to further improve the vignettes. After successful testing, we randomly divided the panel members into 4 groups (Multimedia Appendix 1). The groups were presented with either a life-threatening (high-risk) vignette in the NICU (from now on referred to as the “high-risk case”), or a non–life-threatening (low-risk) vignette regarding RA (referred to as the “low-risk case”). Details of the vignettes are described in Multimedia Appendix 2. The high-risk case described a baby in the NICU that possibly had sepsis. In this case, we asked the panel members to empathize with the role of the baby’s parents. The low-risk case described a patient with RA, and panel members were asked to empathize with the role of the patient. In both situations, the physician made a treatment recommendation. This recommendation was made by a physician only in the control group and by a physician supported by a CDSS application in the intervention group. After reading the vignette, the panel members were asked to fill in the questionnaire. Possible answers were given using a 7-point Likert scale, with 1 indicating “strongly disagree” and 7 indicating “strongly agree.”

**Figure 2:**
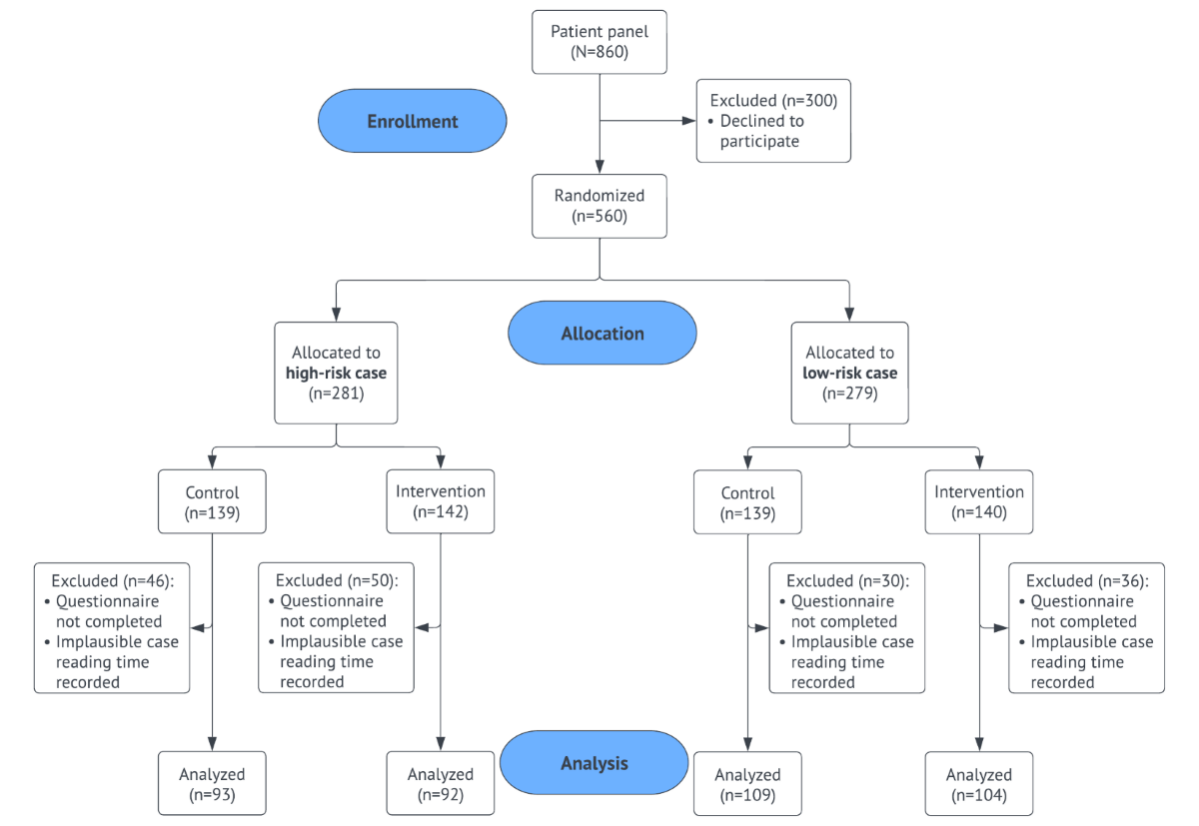
Inclusion procedure of the University Medical Center Utrecht panel members.

We tracked the time it took panel members to read the vignettes. To minimize the potential loss of statistical power due to panel members not being diligent and motivated to complete the survey, we excluded the 2.5% slowest and fastest readers from all analyses. Figure 2 illustrates the patient inclusion and exclusion procedure in a flow diagram.

### Data Analyses

We presented the results of all groups in medians (IQRs), stratified by case. We compared perceived trust between the intervention and control group using the Mann-Whitney *U* test (Wilcoxon rank sum test).

We assessed the presence or absence of effect modification using a two-way multivariate analysis of variance (MANOVA) or multivariate analysis of covariance (MANCOVA), as appropriate. The participant’s sex, age, education level, general trust in health care, and general trust in technology were considered potential effect modifiers in the relation between the use of a CDSS and patient-physician trust. We further stratified the results by sex in case of effect modification to examine this potential effect.

All analyses were performed using SPSS edition 26 (IBM SPSS Inc) [42]. Differences with a 2-sided *P* value of <.05 were considered statistically significant.

### Ethical Considerations

All participants provided informed consent digitally for this study. The panel members received an introductory text with information about this study. At the end of the introductory text, the privacy statement informed the panel members that by clicking the “Continue” button, they agreed to the use of their data in this study. Data were anonymized and IP addresses were not stored.

The questionnaires were administered in Qualtrics in accordance with Utrecht University guidelines and stored on the Yoda server, which is a research data management service from the Utrecht University, among others, enabling secure storage of the research data [43,44]. The methods have been performed based on relevant guidelines and regulations. The institutional review board of UMC Utrecht waived ethical approval because it ruled that this study did not concern a medical research question.

## Results

### Demographics of the Study Population

In total, 398/860 (46.3%) panel members of the UMC Utrecht patient panel participated in this study. Characteristics of the study population can be seen in Table 2. Participants were older in the intervention group of both cases. In the high-risk case, the intervention group consisted of more men (52/92, 56.5%), whereas the control group consisted of more women (54/93, 58.1%). In the low-risk case, both the control and intervention groups consisted of more women (55/109, 50.5% and 56/104, 53.8%, respectively). The education level was similar across the groups. In all groups, the majority of participants (62/93, 66.7%; 60/92, 65.9%; 69/109, 63.3%; and 68/104, 66.0%) had a high education level, that is, a degree from a university of applied sciences or higher.

**Table 2:** Demographics of the study population by case.

Dimension		High-risk case			Low-risk case		
		Control (n=93)	Intervention (n=92)	Control (n=109)			Intervention (n=104)
Age, mean (SD)		57.9 (11.7)	58.9 (12.1)	60.1 (12.0)			61.6 (10.0)
**Sex, n (%)**							
	Men	39 (41.9)	52 (56.5)			54 (49.5)	48 (46.2)
	Women	54 (58.1)	40 (43.5)			55 (50.5)	56 (53.8)
**Education level, n (%)**							
	Lower	31 (33.3)	31 (34.1)			40 (36.7)	35 (34)
	Higher	62 (66.7)	60 (65.9)			69 (63.3)	68 (66)

### Measured Trust in the Physician

Based on the results of the factor analysis and Cronbach α calculation, we assessed trust on both a multidimensional and a unidimensional scale. The reason to use a unidimensional scale was that the factor analysis initially showed 2 factors (Table S1 in Multimedia Appendix 3); however, after removing the 2 items that loaded strongly on both factors and 1 item that primarily loaded on the second factor, only 1 factor remained. The reason for using a multidimensional scale was that the Cronbach α of the 3 dimensions was considered high enough (>0.75) to measure trust on a multidimensional scale (Table S2 in Multimedia Appendix 3).

Overall, trust in the physician was high, with a median of 5.8 (control group IQR 5.0-6.5; intervention group IQR 4.7-6.2) in the high-risk case and 6.0 (control group IQR 5.3-6.5; intervention group IQR 5.1-6.5) in the low-risk case on a 7-point Likert scale (Multimedia Appendix 4). The high-risk case showed a lower median for the integrity of the physician using a CDSS (6.3, IQR 5.3-6.8 vs 6.0, IQR 5.0-6.7; *U*=3590.0; *P*=.06; Table 3) compared to the physician who did not use a CDSS, but these results were not statistically significant. Similarly, perceived competence was lower in the intervention group compared to the control group (median 5.8, IQR 4.8-6.5 vs 5.6, IQR 4.3-6.3; *U*=3771.5; *P*=.16). We observed no between-group differences in perceived competence, integrity, and benevolence of the low-risk group.

In the analyses exploring whether the results were different among subgroups, in the high-risk case, the effect of a CDSS application on the perceived competence of the physician depended on the participant’s sex (*F*_1,181_=4.694; *P*=.03; Multimedia Appendix 5). In women, perceived competence and integrity were significantly lower in physicians who used a CDSS compared to physicians who did not (*U*=740.5; *P*=.009 and *U*=756.0; *P*=.01, respectively; Table 4), whereas no such statistically significant differences were found in men.

In the low-risk case, results showed that the effect of the CDSS application on the perceived benevolence and integrity depended on the participant’s trust in technology in general (*F*_1,209_=6.943; *P*=.009 and *F*_1,209_=4.119; *P*=.04, respectively; Multimedia Appendix 5). An increase in the participant’s trust in technology, in general, led to an increased perceived integrity and benevolence of physicians using a CDSS. This increase was more significant in the intervention group compared to the control group.

**Table 3:** Measured levels of trust of the 3 dimensions between groups, by case.

Dimension	High-risk case					Low-risk case			
	Control (n=93)	Intervention (n=92)	*U* test^a^	*P* value^b^	Control (n=109)		Intervention (n=104)	*U* test	*P* value
Competence, median (IQR)	5.8 (4.8-6.5)	5.6 (4.3-6.3)	3771.5	.16	6.0 (5.3-6.5)		6.0 (5.0-6.5)	5319.0	.43
Integrity, median (IQR)	6.3 (5.3-6.8)	6.0 (5.0-6.7)	3590.0	.06	6.0 (5.5-6.8)		6.0 (5.3-6.8)	5698.0	.95
Benevolence, median (IQR)	5.8 (5.0-6.6)	5.8 (4.9-6.2)	4055.5	.54	6.0 (5.4-6.6)		6.0 (5.3-6.6)	5829.0	.72

^a^*U* test: Wilcoxon test statistic.

^b^The *P* values in the table indicate the difference between the control and intervention groups.

**Table 4:** Measured levels of trust of the 3 dimensions in the high-risk case, stratified by sex.

Dimension	Men					Women				
	Control (n=39)	Intervention (n=52)	*U* test^a^	*P* value^b^	Control (n=54)		Intervention (n=40)	*U* test	*P* value	
Competence, median (IQR)	5.0 (4.5-6.3)	5.9 (4.3-6.4)	1111.0	.43	6.0 (5.2-6.8)		5.4 (4.0-6.0)	740.5	.009	
Integrity, median (IQR)	6.3 (5.3-6.8)	6.1 (5.0-6.8)	1001.0	.92	6.5 (5.3-7.0)		5.8 (5.0-6.3)	756.0	.01	
Benevolence, median (IQR)	5.8 (5.0-6.4)	6.0 (5.1-6.4)	1064.5	.68	5.7 (5.0-6.8)		5.5 (4.8-6.0)	918.5	.21	

^a^*U*: Wilcoxon test statistic.

^b^*P* values indicate the difference between the control and intervention groups.

## Discussion

### Principal Findings

We aimed to assess the extent to which patient-physician trust was affected by using a CDSS application. We found that, in general, trust in physicians was high. Nonetheless, in the high-risk case, we observed that trust, in terms of competence and integrity, was lower in physicians using a CDSS compared to physicians who did not, and that the differences were larger in women than in men. No differences were found in the low-risk case. However, we found that perceived benevolence and integrity in the low-risk case depended on the participant’s trust in technology in general.

### Comparison With Prior Literature

To our knowledge, this study is among the first to examine the impact of AI-based CDSSs on patient-physician trust in both a clinical high-risk and low-risk setting. There are several possible explanations for our results. First, prior to our study, we asked several physicians of the UMC Utrecht about their expectations regarding CDSS applications. Some anticipated that the use of CDSS applications in clinical practice could evoke a critical response from patients, believing that CDSS applications “first must prove their value before being accepted by patients.” Regarding competence, physicians mentioned that the use of a CDSS application could raise doubts about their professionalism, as it could come across as “being dependent on such an application.” This dependence and the associated loss of competence, known as “deskilling,” is a known concern of the introduction of clinical decision support applications in health care [45]. Some of these concerns seem reasonable, as our study indicates that patients’ trust in their physicians can decrease when a CDSS is used. Moreover, the decrease found in trust in terms of the integrity of a physician who uses a CDSS could be explained by concerns about data protection. Patients may feel they have no control over what happens with their personal data. However, some studies indicated that privacy was not an issue for some patients, as long as they trusted their physician [46,47]. Yakar et al [48] studied the general population’s view on the use of AI in health care through a web-based survey in the Netherlands and found less trust in AI than initially hypothesized. They, and some others, also found that women were less trusting of AI than men [48,49]. A reason for this could be that the women participating in this study were aware of the potential presence of gender bias in AI, as this issue has often been raised in previous research and media [50-52]. Additionally, women generally are considered to have more risk aversion than men [53]. Both these reasons combined could explain why the effect of using a CDSS on the perceived trust in the physician in the high-risk case depended on the participant’s sex and why this was not the case in the low-risk case.

In the low-risk case, our results showed that a patient’s general trust in technology played a significant role in the effect that CDSS applications could have on the perceived integrity and benevolence of the physician. An increase in the perceived integrity and benevolence of the physician seemed to be more likely in patients who had more trust in technology in general. This is in line with previous research investigating the association between trust in technology in general and trust in the implementation of AI in medicine [48,54].

### Implications

Results from this study underline that mainly communicating about the technological aspects of AI algorithms in health care may ultimately hamper the successful implementation and acceptance of these algorithms in clinical practice. Personalized health care starts with putting the patient’s values first. Therefore, we believe that the development of AI-based CDSSs in health care should be transparent in terms of methods, alternatives, and understandability. In addition, patients should be involved early in the developmental phase of the CDSS, and the use of AI should be explained well during the visit with the health care professional. Furthermore, AI applications should not only be developed to predict the prognosis of a certain disease (precision medicine) but also to improve and facilitate discussions between patients and physicians about the patient’s illness and personal care needs (shared decision-making). The discussion between the patient and the physician may be improved by being transparent about the CDSS application. This potentially leads to more trust in the application, which, as shown in this study, could lead to an increase in the perceived integrity and benevolence of the physician. Moreover, it seems sensible to distinguish between the types of situations or patient populations in the development of AI algorithms for CDSS in health care; the results from our study indicate that less favorable reactions occur when AI is used in more life-threatening situations, which could also be due to the different patient populations, for example, babies in an acute situation versus adults in a chronic situation. By implementing AI-based CDSS in less risky situations first, patients could get acquainted with such CDSSs before implementing them in high-risk situations. Further study is warranted to establish the best implementation strategy regarding AI-based CDSSs in clinical care.

We recommend replicating this study while providing a clear explanation of the AI application. For example, by explaining how it works, how patient data are handled, and the benefits of such AI-based CDSSs for the patients. In addition, further research with a larger sample size and in different preventive care and cure settings, and across several age groups, should be conducted to confirm the results found in this study. External validation of our results will inform us whether our results are similar in, for example, patients in other health care settings or areas. Additionally, qualitative research methods should be considered to gain more insights into the reasons patient-physician trust might decline when using a CDSS from a patient perspective.

### Strengths and Limitations

Vignette studies have been criticized in the past for their limitations. First, a frequently heard criticism of vignette studies is the lack of reality due to their hypothetical nature [55]. However, we minimized this by creating both hypothetical cases in collaboration with physicians working in the involved departments—the neonatology and rheumatology departments—and tested whether the participants could empathize well with the situation during a pilot study. Second, the participants in this study were (or still are) patients of the UMC Utrecht. This made it easier for the participants to empathize with the vignettes because they may have been in similar situations themselves, and they indicated they could (median 6.0, IQR 5.0-7.0 in all 4 groups). Third, it has been shown that participants of vignette studies are not always diligent and motivated to complete surveys or take these kinds of experiments seriously, which may reduce statistical power [56]. Participants could, for example, have put the questionnaire aside for a while, or might not have read the described situations properly [57]. We reduced this effect by tracking the time the participants took to read the vignettes and by excluding the fastest and the slowest 2.5% of readers from the analyses. Therefore, participants spending a remarkably long or short time reading the vignette were not included.

### Conclusions

Trust in physicians is generally high among patients. However, our findings point toward a negative effect that AI applications can have on the patient-physician relationship, especially among women. We, therefore, believe that, for a successful adoption of AI applications in clinical practice, patients should be involved in both the development and implementation of such applications. Moreover, a broader societal discussion needs to take place about humane values and AI to gain insights into how we want AI to influence our lives when we encounter health care. In addition, clear communication to patients and society about the functions of the AI applications and what personal data they use seems equally important.

## Appendix

Multimedia Appendix 1 Study design.

Multimedia Appendix 2 Details of the high-risk and low-risk vignettes.

Multimedia Appendix 3 Questionnaire factor analysis and Cronbach α.

Multimedia Appendix 4 Unidimensional trust scores, stratified by case.

Multimedia Appendix 5 Multivariate regression analyses to test potential effect modification.

## Abbreviations

ADAM: Applied Data Analytics in Medicine
AI: artificial intelligence
CDSS: clinical decision support system
MANCOVA: multivariate analysis of covariance
MANOVA: multivariate analysis of variance
NICU: Neonatal Intensive Care Unit
RA: rheumatoid arthritis
STROBE: Strengthening the Reporting of Observational Studies in Epidemiology
UMC: University Medical Center
