# Supplementary material for: The Effect of Artificial Intelligence on Patient-Physician Trust: Cross-Sectional Vignette Study
Source: J Med Internet Res. 2024 May 28;26:e50853. doi: 10.2196/50853 (PMC11167322; doi:10.2196/50853)
Supplement: Multimedia Appendix 1 [file jmir_v26i1e50853_app1.doc]

# Multimedia Appendix 1

## Study design

Table 1. Design of the study: random allocation of panel members across the four groups.

| **Project** | **Control (no CDSS)** | **Intervention (with CDSS)** |
| --- | --- | --- |
| NICU | Control group 1 (N = 93) | Intervention group 1 (N = 92) |
| RA | Control group 2 (N = 109) | Intervention group 2 (N = 104) |
