# Supplementary material for: The Effect of Artificial Intelligence on Patient-Physician Trust: Cross-Sectional Vignette Study
Source: J Med Internet Res. 2024 May 28;26:e50853. doi: 10.2196/50853 (PMC11167322; doi:10.2196/50853)
Supplement: Multimedia Appendix 2 [file jmir_v26i1e50853_app2.doc]

# Multimedia Appendix 2

## High-risk vignette, translated from Dutch to English

### Vignette of the control group

Imagine the following situation:

After 27 weeks of pregnancy, you gave birth to your baby Lucas in the hospital. This is extremely early, which is why Lucas has been in an incubator in the neonatal intensive care unit for two weeks now. Due to the early birth, Lucas is at risk of dying from complications. You have been at his bedside day and night for the past few weeks.

Lucas’ treating physician comes to you and tells you that there are doubts about Lucas’ health. The physician says that Lucas is at risk of blood poisoning (sepsis), which can be fatal without proper treatment. To treat blood poisoning, antibiotics can be administered to Lucas. The physician takes blood from Lucas to see if he has any infections. However, the final result of the blood test will **only be available in three days**. That may be too late.

The physician **is in doubt** about whether antibiotics should be administered to Lucas now. Antibiotics can have very serious side effects. By giving antibiotics, Lucas can become resistant and, as a result, he may not be able to be treated for an infection later on. This can be fatal in the worst case. That is why the physicians tries to administer as few antibiotics as possible.

Have you read the above text? Then click on “continue”.

--- page split ---

The physician tells you that he/she does **not recommend giving any antibiotics** to Lucas yet, because at the moment the physician does not think the risk of blood poisoning is high enough.

There is a risk associated with the physician’s recommendation. If the physician makes the wrong risk assessment, it could mean that Lucas’s situation will worsen. In the worst case this can also lead to death.

Important to know about the physician’s recommendation is:

- The physician takes into account, among other things, Lucas’s changed temperature, blood pressure and heart rate.
- The physician has experience with these kinds of situations and uses this experience to make a prediction.

### Vignette of the intervention group

After 27 weeks of pregnancy, you gave birth to your baby Lucas in the hospital. This is extremely early, which is why Lucas has been in an incubator in the neonatal intensive care unit for two weeks now. Due to the early birth, Lucas is at risk of dying from complications. You have been at his bedside day and night for the past few weeks.

Lucas’s treating physician receives an alarm of a **computer system** because Lucas shows abnormal values. Then, the physician comes to you and tells you that there a doubt about Lucas’ health. The physician says that Lucas is **at risk** of blood poisoning (sepsis), which can be fatal without proper treatment. To treat blood poisoning, antibiotics can be administered to Lucas. The physician takes blood from Lucas to see if he has any infections. However, the final result of the blood test will **only be available in three days**. That may be too late.

The physician **is in doubt** about whether antibiotics should be administered to Lucas now. Antibiotics can have very serious side effects. By giving antibiotics, Lucas can become resistant and, as a result, he may not be able to be treated for an infection later on. This can be fatal in the worst case. That is why the physicians tries to administer as few antibiotics as possible.

Have you read the above text? Then click on “continue”.

--- page split ---

The physician looks into a computer system and tells you that he/she **does not recommend giving any antibiotics** to Lucas yet, because the physician sees that the risk of blood poisoning at the moment is not high enough.

There is a risk associated with the physician’s recommendation. If the physician makes the wrong risk assessment, it could mean that Lucas’s situation is worsening. In the worst case this can also lead to death.

Important to know about the physician’s recommendation is:

- The physician receives clinical decision support from a **computer system** predicting Lucas’s risk of blood poisoning based on information on temperature, blood pressure and heartrate combined.
- It is an advanced computer system that combines medical information to arrive at a risk prediction.

## Low-risk vignette, translated from Dutch to English

### Vignette of the control group

Imagine the following situation:

Five years ago, you started to suffer from rheumatic symptoms in your joints, mainly in your knees. Every three months you visit the rheumatologist in the hospital to discuss the course of your disease and to discuss your medication. You are taking medication based on advice of your physician.

These drugs ensure that you no longer have rheumatic flares. During a rheumatoid flare you experienced severe burning pains in your knees. These flares could last for weeks and prevented you from functioning normally in daily life. However, the drugs that you take have serious side effects: you regularly suffer from headaches, dizziness, and vomiting.

Have you read the above text? Then click on “continue”.

--- page split ---

Your physician **recommends** you to **lower your medication dose**, because your physician thinks that with less medication you will still have a low risk of a flare. That means the side effects of the medication will likely decrease.

On the other hand, if the physician makes the wrong risk assessment, it could mean that you will suffer from flares again. Resulting in not being able to properly function for weeks because of severe pain.

Important to know about your physician’s recommendation is:

- The physician will do a number of tests on your knees, take into account the results of blood tests taken from you and feel for any swelling. Based on this, your physician thinks your risk of flares has decreased.
- The physician has experience with these kinds of situations and uses this experience to make a prediction.

### Vignette of the intervention group

Five years ago, you started to suffer from rheumatic symptoms in your joints, mainly in your knees. Every three months you visit the rheumatologist in the hospital to discuss the course of your disease and to discuss your medication. You are taking medication based on advice of your physician.

These drugs ensure that you no longer have rheumatic flares. During a rheumatoid flare you experienced severe burning pains in your knees. These flares could last for weeks and prevented you from functioning normally in daily life. However, the drugs that you take have serious side effects: you regularly suffer from headaches, dizziness, and vomiting.

Have you read the above text? Then click on “continue”.

--- page split ---

Your physician looks into a computer system in which the physician can see that with less medication, you still have a low risk of a flare. Therefore, the physician **recommends** you to **lower your dose of medication**. Meaning that side effects of the medication will likely decrease.

On the other hand, if the physician makes the wrong risk assessment, it could mean that you will suffer from flares again. Resulting in not being able to properly function for weeks because of severe pain.

Important to know about your physician’s recommendation is:

- Your physician receives clinical decision support from a **computer system** that predicts your flares based on information about your disease progress over the years.
- It is an advanced system that combines medical information to arrive at a risk prediction.
