# Supplementary material for: The Effect of Artificial Intelligence on Patient-Physician Trust: Cross-Sectional Vignette Study
Source: J Med Internet Res. 2024 May 28;26:e50853. doi: 10.2196/50853 (PMC11167322; doi:10.2196/50853)
Supplement: Multimedia Appendix 3 [file jmir_v26i1e50853_app3.doc]

# Multimedia Appendix 3

## Questionnaire factor analysis and Cronbach α

Table S1. Explorative factor analysis with in bold the items that either loaded on both factors or only on the second factor and, therefore, were removed.

|  | **Factor** | |
| --- | --- | --- |
| **scale-items** | **1** | **2** |
| I trust this physician’s recommendation will put my medical needs above other interests | **.494** | **.409** |
| I feel that with this recommendation the physician does everything to help me. | .814 | .174 |
| This physician really cares about me as a person | .769 | .137 |
| This physician’s recommendation takes into account my wishes and puts them first | .635 | .212 |
| This physician is a real expert in taking care of medical problems like mine | .843 | -.020 |
| I trust this physician’s judgement about this medical problem | .961 | -.104 |
| I trust this physician’s recommendation and therefore have no need for a second opinion | .926 | -.308 |
| I trust my physician’s recommendation so much that I will follow it | .979 | -.235 |
| I trust this physician will tell me if he/she makes a mistake in this recommendation | .729 | -.029 |
| If this physician tells me this recommendation, then I also believe that this recommendation is correct | .917 | -.034 |
| I feel that this physician keeps the medical information used for this recommendation private | **.055** | **.790** |
| I feel that this physician is honest with me. | .777 | .255 |
| I feel like this physician is not holding anything back from me. | .785 | .193 |
| I trust that this physician will treat my medical information confidentially | **.543** | **.479** |
| This physician’s recommendation is in my personal best interest | .707 | .236 |
| Eigenvalues | 9.646 | 1.069 |
| Explained variance (%) | 64.31 | 7.13 |

Table S2. Cronbach α.

We used the Cronbach α to measure the internal consistency of each dimension, providing the overall reliability for a set of items (questions).^34^ A Cronbach α of more than .75 is considered as high.^47^

| **Dimension** | **Cronbach α** | | **Items** |
| --- | --- | --- | --- |
| Benevolence | | .90 | 5 |
| Competence | | .92 | 4 |
| Integrity | | .93 | 4 |

*Notes:* α = alpha.
