# Supplementary material for: The Effect of Artificial Intelligence on Patient-Physician Trust: Cross-Sectional Vignette Study
Source: J Med Internet Res. 2024 May 28;26:e50853. doi: 10.2196/50853 (PMC11167322; doi:10.2196/50853)
Supplement: Multimedia Appendix 4 [file jmir_v26i1e50853_app4.doc]

# Multimedia Appendix 4

## Trust measured unidimensional

Table 1. Unidimensional trust scores, stratified by case.

|  | **High-risk case** | | **Low-risk case** | |
| --- | --- | --- | --- | --- |
|  | Control | Intervention | Control | Intervention |
| Median (IQR) | 5.8 (5.0-6.5) | 5.8 (4.7-6.2) | 6.0 (5.3-6.5) | 6.0 (5.1-6.5) |

*Notes:* IQR = interquartile range.
