# Supplementary material for: The Effect of Artificial Intelligence on Patient-Physician Trust: Cross-Sectional Vignette Study
Source: J Med Internet Res. 2024 May 28;26:e50853. doi: 10.2196/50853 (PMC11167322; doi:10.2196/50853)
Supplement: Multimedia Appendix 5 [file jmir_v26i1e50853_app5.doc]

# Multimedia Appendix 5

## Multivariate regression analyses

Table 1. Multivariate regression results to test potential effect modification.

|  | **High-risk case** | | **Low-risk case** | |
| --- | --- | --- | --- | --- |
|  | *F* test (*df*) | *P* value | *F* test (*df*) | *P* value |
| Trust in healthcare in general |  |  |  |  |
| - Benevolence | 1.523 (1, 181) | .22 | 0.080 (1, 209) | .78 |
| - Integrity | 1.793 (1, 181) | .18 | 0.241 (1, 209) | .62 |
| - Competence | 1.626 (1, 181) | .20 | 0.155 (1, 209) | .69 |
| Trust in technology in general |  |  |  |  |
| - Benevolence | 1.531 (1, 181) | .22 | **6.943 (1, 209)** | **.009** |
| - Integrity | 2.610 (1, 181) | .11 | **4.119 (1, 209)** | **.04** |
| - Competence | 0.836 (1, 181) | .36 | 2.037 (1, 209) | .16 |
| Education |  |  |  |  |
| - Benevolence | 1.981 (1, 180) | .16 | 0.879 (1, 208) | .35 |
| - Integrity | 1.212 (1, 180) | .27 | 1.180 (1, 208) | .28 |
| - Competence | 0.446 (1, 180) | .51 | 0.011 (1, 208) | .92 |
| Age |  |  |  |  |
| - Benevolence | 0.077 (1, 181) | .78 | 0.232 (1, 209) | .63 |
| - Integrity | 0.150 (1, 181) | .70 | 0.129 (1, 209) | .72 |
| - Competence | 0.050 (1, 181) | .82 | 0.001 (1, 209) | .98 |
| Sex |  |  |  |  |
| - Benevolence | 0.073 (1, 181) | .79 | 0.014 (1, 209) | .91 |
| - Integrity | 0.566 (1, 181) | .45 | 0.072 (1, 209) | .79 |
| - Competence | **4.694 (1, 181)** | **.03** | 0.028 (1, 209) | .87 |

*Notes: F* = value indicating the variation in size between the between-groups and the within-groups variance; *df* = degrees of freedom; *P* = significance (*P*-) value indicates the significance of the interaction term.
